# Social support resources in adolescents and young adults with advanced cancer: a qualitative analysis

**DOI:** 10.1186/s12904-024-01527-y

**Published:** 2024-07-31

**Authors:** Nancy Lau, Angela Steineck, Casey Walsh, Kaitlyn M. Fladeboe, Joyce P. Yi-Frazier, Abby R. Rosenberg, Krysta Barton

**Affiliations:** 1 Center for Child Health, Behavior and Development, Seattle Children’s Research Institute, 1920 Terry Ave, Seattle, WA 98101 USA; 2 Department of Psychiatry and Behavioral Sciences, University of Washington School of Medicine, Seattle, WA USA; 3 MACC Fund Center for Cancer and Blood Disorders, Department of Pediatrics, Medical College of Wisconsin, Milwaukee, WI USA; 4 Clinical Research Division, Fred Hutchinson Cancer Center, Seattle, WA USA; 5 Ben Towne Center for Childhood Cancer Research, Seattle Children’s Research Institute, Seattle, WA USA; 6 Department of Pediatrics, University of Washington School of Medicine, Seattle, WA USA; 7 Department of Psychosocial Oncology and Palliative Care, Dana-Farber Cancer Institute, Boston, MA USA; 8 Department of Pediatrics, Boston Children’s Hospital, Boston, MA USA; 9 Department of Pediatrics, Harvard Medical School, Boston, MA USA; 10 Biostatistics Epidemiology and Analytics for Research (BEAR) Core, Seattle Children’s Research Institute, Seattle, WA USA

**Keywords:** Social support, Qualitative research, Psychosocial support systems, Adolescents, Young adults, Advanced cancer

## Abstract

**Purpose:**

Adolescents and Young Adults (AYAs) with cancer are an at-risk group with unique palliative and supportive care needs. Social support in AYAs with cancer is associated with better coping, quality of life, and psychosocial well-being. Here, we extend existing research to examine the sources and types of support received by AYAs with advanced cancer.

**Methods:**

AYAs participated in a semi-structured, 1:1 interview on communication and psychosocial support needs. The present analysis focused on social support experiences for AYAs with advanced cancer. Directed content analysis was used to develop the codebook. Established social support constructs provided a coding framework. We presented our qualitative findings as a code frequency report with quantified frequency counts of all “source of support” and “type of support” codes. We assigned a global “sufficiency of support code” to each AYA.

**Results:**

We interviewed 32 AYAs with advanced cancer (M*age* = 18, SD*age* = 3.2, 41% female). Most AYAs identified family (namely, caregivers) as their primary source of support and stated that family universally provided all types of support: emotional, informational, instrumental, and social companionship. They received informational and emotional support from clinicians, and received emotional support and social companionship from healthy peers, cancer peers, and their existing community. One-third of participants were coded as having “mixed support” and described a lack of support in some domains.

**Conclusion:**

AYAs with advanced cancer described caregivers as their universal source of support, and that other support sources provided support for specific needs. Future research should continue to evaluate social support needs and family-based palliative and supportive care interventions to bolster social support resources in this high-risk group.

**Supplementary Information:**

The online version contains supplementary material available at 10.1186/s12904-024-01527-y.

## Background

Adolescents and Young Adults (AYAs) with cancer are an at-risk group with unique palliative and supportive care needs [1]. AYAs with cancer experience worse health-related quality of life and psychosocial outcomes than their younger pediatric and older adult counterparts [2]. Among other negative psychosocial outcomes, AYAs with cancer experience depression, anxiety, and stress [3, 4]. Cancer disrupts social and developmental transitions towards independence from parents and establishment of romantic relationships. Social isolation and loneliness in AYAs with cancer has been associated with worse physical functioning, fatigue, pain, anxiety, and depression [5]. Theoretical models of social support posit that social support buffers against the negative effects of stressful life events on health through perceived support (i.e., beliefs in availability of support) and actual help received [6]. A social capital framework for palliative care may provide a roadmap for identifying social contexts that have a positive or negative impact on end-of-life care [7].

Social support is associated with adaptive coping strategies, better adjustment to cancer, and improvements in psychological distress in AYAs with cancer [8, 9]. In AYAs with cancer, greater perceived support from family, friends, and healthcare providers has been associated with better global mental health [10]. Findings from a qualitative study of AYAs within 2 years of their first cancer diagnosis found that parents were the primary source of emotional support (i.e., empathy and supportive coping), informational support (i.e., advice, guidance, and problem-solving), and instrumental support (i.e., material, physical, functional assistance), even for older AYAs who had romantic partners and children [11]. Cancer peers provided informational and emotional support, while healthy peers provided opportunities for distraction [11]. Healthcare providers provided informational support and emotional support [11]. Other research studies have shown that AYA cancer patients receive social support from > 1 individual, with emotional and instrumental support being the most common, and that social support supplements individual coping resources [12, 13].

The current qualitative analysis expands upon previous research to AYAs with advanced cancer, a particularly high-risk group with relapsed/recurrent, refractory, or metastatic disease or are unlikely to be cured. For AYAs with advanced cancer, social support needs may be even greater due to frequent or extended hospitalizations, travel or relocation for treatment, prognostic uncertainty, repeated life disruptions due to cancer recurrence, and the psychological impact of navigating a life-threatening disease [14–17]. AYAs with advanced cancer have additional unmet palliative and supportive care needs such as prognostic uncertainty, communication about end-of-life care, and that death may be a consequence of their disease [14, 15]. There has been one survey-based study to date that has examined social support in young adults with advanced cancer that utilized a consistent definition of advanced cancer but was with an older cohort of young adults with cancer ages 20 to 40. This study found that higher perceived total social support was associated with greater psychological quality of life, existential quality of life, and less grief. The Interpersonal Support Evaluation List subscale of: “appraisal support” was associated with greater psychological quality of life and existential quality of life, and less grief; “tangible support” was associated with greater psychological quality of life and existential quality of life; and, “belonging support” was associated with better existential quality of life [18]. This suggests that analyses of specific types of support received by AYAs with advanced cancer may be additive and informative. We aimed to expand on the relative paucity of research on social support in AYAs with advanced cancer in the current qualitative analysis. Our main research question was: Who provides what type(s) of social support to AYAs with advanced cancer?

## Methods

### Design, setting, and participants

This secondary analysis utilized data from all AYA interviews conducted as part of the “Exploring the Concept of a ‘Good Death’ Study”. This study was approved by the Seattle Children’s Hospital (SCH) Institutional Review Board, and has been described in detail elsewhere [19–21]. Eligible participants were pediatric AYAs (ages 14–25) with advanced cancer ≥ 2 weeks prior to enrollment. Although the National Cancer Institute definition of AYAs includes ages 15–39, we recruited for a narrower age range of pediatric AYAs typically treated in pediatric cancer centers such as Seattle Children’s Hospital where this study was conducted. Advanced cancer was defined as relapsed/recurrent disease; refractory disease at any time during treatment; eligible for a phase I clinical trial; enrolled in hospice or palliative care; or, patients with < 50% likelihood of cure as judged by their oncologist. The study coordinator reviewed clinic lists to identify those with upcoming clinic visits who met study inclusion criteria. The study coordinator then asked a member of the oncology team for permission to approach and query potential participants about interest in the study. Patients and parents were approached in a private room in the oncology unit. We purposely recruited individuals from the following age strata: patients aged 14–17 years (early adolescence) and aged 18–25 (late adolescence/early adulthood) for representation of different developmental stages. Patients were excluded from the study if they were unable to speak/read/write English; were cognitively or physically unable to participate; or, due to parental refusal for patients < 18 years-old. Written informed consent was obtained for those ≥ 18 years-old, and assent/parental consent for those ages 14–17 years-old.

### Research team

Authors’ backgrounds included health services research (NL, AS, CW, JYF, ARR, KSB), implementation science (NL), psychology (NL, KMF, JYF), social work (CW), bioethics (ARR, KSB), pediatric oncology (AS, ARR), psycho-oncology research (NL, AS, CW, KMF, JYF, ARR, KSB), and palliative care research (NL, AS, JYF, ARR, KSB).

### Qualitative data

We followed the Standards for Reporting Qualitative Research (SRQR) guidelines for complete reporting of qualitative research data collection and analysis procedures [22]. A PhD-trained qualitative methodologist (KSB) with no prior relationship to participants conducted semi-structured, 1:1 interviews from December 2017–September 2018. No caregivers were present during patient interviews. Based on patient preferences, interviews were conducted in-person or by phone, and audio-recorded, transcribed verbatim, and de-identified. Interviews were 20–75 min long (average = 37 min). Interviewer memoed during the data collection process. Interviews were conducted at a single cross-sectional timepoint. The interview guide covered topics on family communication and psychosocial support needs for AYAs with advanced cancer (Appendix A). As part of the interview, all participants were asked: Who has helped you the most during this time?; Is there anything that you feel like [support person] says or does that is particularly helpful?; Who else supports you?; Are there any supports/conversations/information you feel like you do not have and would like to have?; When things get hard, what helps you most?; Do you have a support person, group or community to reach out to if you feel anxious or sad?

### Data analyses

We utilized directed content analysis [23] to develop a social support codebook based on established social support frameworks [24, 25]. Sources of support (cancer community; existing community; family; peers, providers) and type of support (emotional; informational; instrumental; social companionship) were coded. Types of support were determined and defined based on established social support constructs [26–28]. Emotional support is engagement by others to support coping through active listening, displays of love, care, concern, and empathy. Informational support is giving advice or providing guidance, also to facilitate problem-solving. Instrumental support is concrete aid in the form of material, physical, or functional assistance. Social companionship is providing company for a variety of activities and hospital visits, and valuing being together. A global “sufficiency of support” code was assigned to each participant based on their perceived social support at the time of the study interview. Perceived social support was categorized as Sufficient (endorses feeling supported, being satisfied with support, and experiencing positive social support relationships); Mixed (endorses both feeling supported and unsupported/absence of support in different domains (i.e., sources or types of support); Insufficient (endorses feeling unsupported, absence/lack of support, social isolation, or negative social support relationships); or, Unable to determine. The codebook was refined iteratively by coding waves of transcripts in sets of five, with each wave of coding compared between coders to create clear and concise coding definitions. Interview transcripts were managed and analyzed using DeDoose software (SocioCultural Research Consultants, LLC; www.dedoose.com). Two coders (NL, KSB) coded all transcripts and met twice monthly for consensus conversations. We presented our qualitative findings as a code frequency report [29, 30] (i.e., quantified frequency counts of all “source of support” and “type of support” codes), an established qualitative approach to illustrate and compare types of support received and by which support sources. Findings were then shared with the research team to synthesize linkages between source of support and type of support, and areas in which support was inadequate/lacking. We did not return transcripts to participants or present findings to participants for member checking.

## Results

Participants were 32 AYAs with advanced cancer [M_age_ = 18, SD _age_ = 3.2, range 14–25 years] (Demographics, Table 1). Only two patients who were approached for the study declined to participate, both of whom were in the older adolescent/early adult stratum aged 18–25. Acute Lymphoblastic Leukemia (ALL) was the most common diagnosis (41%). About half of the sample was male (59%) and self-identified as White/Caucasian (56%). Specifically, in response to the interview question, “Who do you feel has helped you the most during this time?,” the majority of participants (94%) identified family as their primary source of support. Of those participants who identified family as their primary support, 90% named their “parents”, or specifically named their “mom” (53% of participants). Two participants (6%) identified peers as their primary source of support. In addition to primary sources of support, participants went on to describe multiple other sources of support. We selected exemplar quotes of the most frequent types of social support provided by specific sources of support (Table 2).

**Table 1:** Participant demographic characteristics (*N* = 32)

Participants	n (%)
Sex	
Male	19 (59)
Female	13 (41)
Diagnosis	
Acute Lymphoblastic Leukemia	13 (41)
Lymphoma	5 (16)
Brain Tumor	4 (13)
Acute Myeloid Leukemia	3 (9)
Sarcoma	3 (9)
Other	4 (13)
Race	
White/Caucasian	18 (56)
Asian	5 (16)
More than one race	5 (16)
Unknown	4 (12)
Ethnicity	
Not Hispanic or Latinx	22 (69)
Hispanic or Latinx	6 (19)
Unknown	4 (12)

**Table 2:** Exemplar quotes of most frequent types of social support provided by specific sources of support

		**Type of Support**	**Representative Quotes**
**Source of Support**	**Family**	Emotional Support	I’m not really sure a certain type of conversation takes place, but it’s more like there’s a reassurance, just kind of a look, like, We’re here for you no matter what, we’ve got you, “from my parents to me. .... And it’s just ... they know that I love them more than anything in the world, and vice versa, I know that they love me unbelievably.” (AYA-5032, 25 year-old, Female)
		Informational Support	“I like to know all the information. To know exactly what’s going on and how to best choose. My mom also does a lot of research and then we talk about it and figure it out together.” (AYA-5034, 15 year-old, Female)
		Instrumental Support	“I’d have to fall back on my mom again, who has driven me to and from the hospital countless times, who has kept track of all my medications for me, who’s looked for different symptoms and taken my temperature and called the hospital and talked with doctors ... and the housekeeping behind treatment that you don’t really think about. ... And it’s been really nice to lean on my mom as what I call my secretary for my medical treatment.” (AYA-5028, 19 year-old, Male)
		Social Companionship	“Probably my Mom and my Dad … and always being there in the hospital and stuff like that.” (AYA-5030, 15 Year-old, Male)
	**Clinicians**	Emotional Support	“Doctors back home have actually...and it’s a smaller facility than here so it’s... You get to know who you’re working with closer, nurses, doctors, physicians. And through that it just feels like I can trust them more. And so the bond is better.” (AYA-5036, 21 year-old, Male)
		Informational Support	“And they definitely have an attitude of no question is a dumb question, because they know how overwhelming it is and they’ve taken several, many years of their life to get to the place where they are in practicing such a high level in the medical field. And there’s just a lot of information. And so, they are totally willing to answer the silliest or little questions. And that’s been a helpful attitude to work with.” (AYA-5028, AYA-5028, 19 year-old, Male)
	**Peers**	Emotional Support	“And then my friends group are wonderful as well, because there’s some sort of comfort that you get from talking with people your age that you can’t get otherwise. And being able to talk with my friends about certain issues and discuss things openly has been helpful.” (AYA-5028, 19 year-old, Male)
		Social Companionship	“Usually like to hang out with friends or something that... That always cheers me up. Or play video games or something like that. Just spend time with friends mostly.” (AYA-5030, 15 Year-old, Male)
	**Existing Community**	Emotional Support	“I ended up joining a female Bible study there, and yeah, and went there every week for a sermon and worship, like church, and yeah, it was great. It just sucks that I just can’t be around that many people that I don’t know if they’re sick or not, with my immune system and stuff right now. But I do know that they’re all, a lot of them, are praying for me, and that means the world to me.” (AYA-5032, 25 year-old, Female)
		Social Companionship	“It’s an organization that’s a....called the [De-identified national organization]. … that’s where I met all of the people that have the same thing as me. They are all athletes. ... Seeing other kids like me … doing the sports that I didn’t think I could ever do when I got amputated. It’s introduced me to wheelchair basketball and I really like that. It’s brought me new experiences and stuff.” (AYA-5030, 15 Year-old, Male)
	**Cancer Community**	Emotional Support	“Getting in touch with the people from my various support groups and telling them what’s on my mind and tell me some of their experiences of what they’re going through with their cancer and things like that are just nice to hear other people who know what you’re going through, when you have cancer you are definitely very alone. ... Speaking as a cancer patient it definitely fills me with hope when somebody can say “yeah, I’ve been there.” (AYA-5013, 24 year-old, Male)
		Social Companionship	“It’s been nice because we’ve been able to communicate with each other, and when we were in the hospital, we got to become friends with each other. It makes things a little more easier to know that you’re not the only person going through it. Other people can be too.” (AYA-5027, 14 year-old, Female)

### Code frequency report

**Fig. 1:**
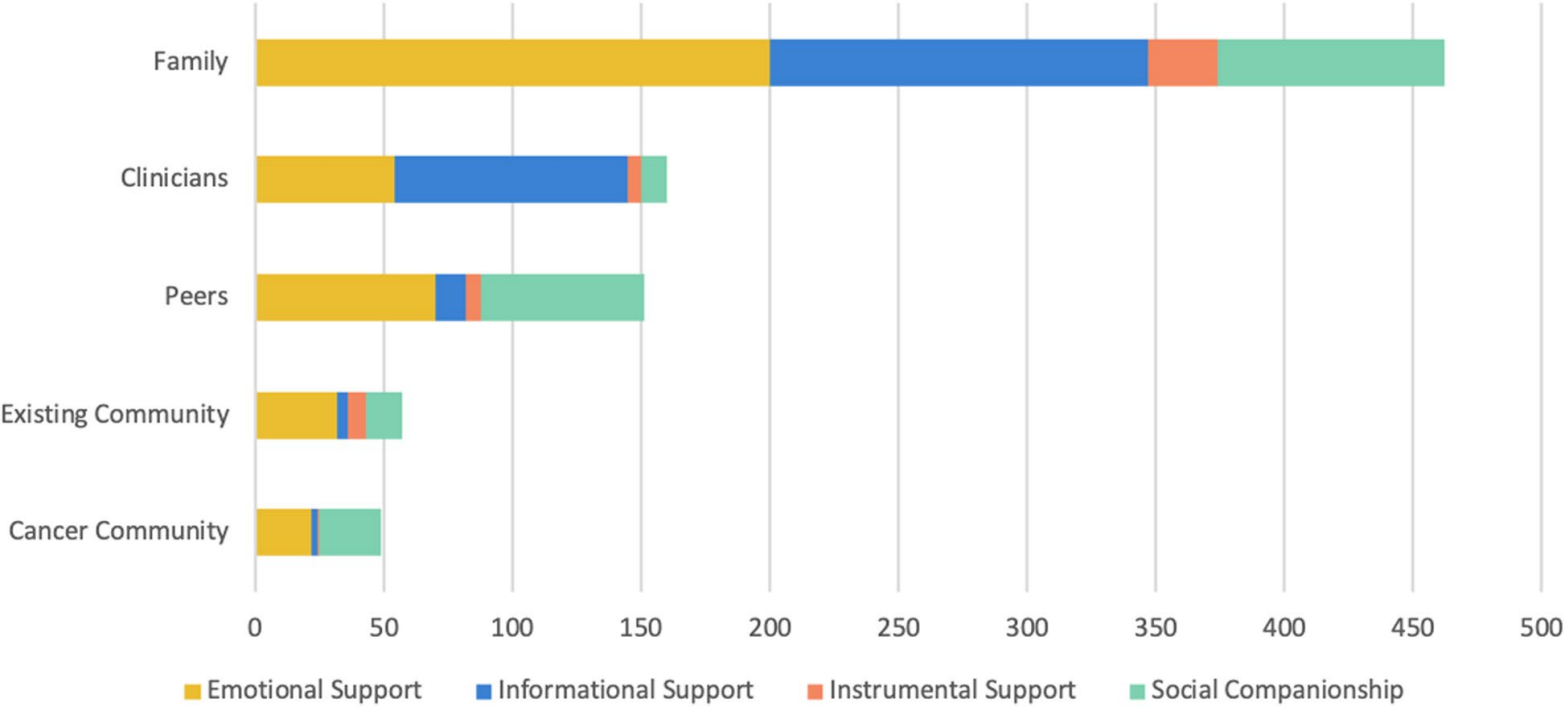
Code frequency counts for types of social support provided by specific sources of support

We classified code frequency counts for types of social support provided by specific sources of support (Fig. 1). In terms of “source of support” code frequency counts, family was the most commonly cited source of support (52.56% of all source of support codes, coded 462 times/879 total), followed by clinicians (18.20% of all source of support codes, coded 160 times/879 total), peers (17.18% of all source of support codes, coded 151 times/879 total), existing community (6.48% of all source of support codes, coded 57 times/879 total), and cancer community (5.57% of all source of support codes, coded 49 times/879 total).

Family (mainly their parents) universally provided all types of support: emotional support (52.91% of all emotional support codes, 200/378), informational support (57.42% of all informational support codes, 147/256), social companionship (44.22% of all social companionship codes, 88/199), and instrumental support (58.70% of all instrumental support codes, 27/46). AYAs described emotional support from family members as affirmations of love and providing reassurance. In terms of informational support, AYAs consistently stated that parents knew as much about their diagnosis and treatment as they did, oftentimes more. The majority of AYAs partnered with their parents in all conversations with clinicians and approached medical decision-making “as a team”. Family members also provided social companionship, “always being there in the hospital”. For AYAs who had to relocate to the treating hospital, a parental caregiver accompanied them, and they were separated from other family members. In addition, parental caregivers were described as the primary source of instrumental support, “what I call my secretary for my medical treatment”.

Clinicians provided the following types of support: informational support (35.55% of all informational support codes, 91/256), emotional support (14.29% of all emotional support codes, 54/378), social companionship (5.03% of all social companionship codes, 10/199), and instrumental support (10.87% of all instrumental support codes, 5/46). Clinicians primarily provided informational support. AYAs did not refer to clinicians with specificity and commonly referenced their “medical team”, “doctor”, or “therapist”. AYAs described their medical team as competent, knowledgeable, and willing to answer questions from the patient and family members. Clinicians were described as having open communication with AYAs and their family members, and that they provided guidance on medical decisions. Clinicians were also described as providing emotional support, mainly in the form of a patient’s trust in their medical team with longstanding relationships forged over time.

Peers provided the following types of support: emotional support (18.52% of all emotional support codes, 70/378), social companionship (31.66% of all social companionship codes, 63/199) informational support (4.69% of all informational support codes, 12/256), and instrumental support (13.04% of all instrumental support codes, 6/46). Peers primarily provided emotional support and social support. They relied on existing friendships from pre-cancer diagnosis, and five participants named a “boyfriend” or a “girlfriend”. In interactions with peers and romantic partners, AYAs strived for normalcy and wanted to engage in developmentally appropriate experiences not centered around their cancer diagnosis or treatment (e.g., “hanging out”, spending time with friends, playing video games, going to the movies, texting).

Existing community and cancer community were the least frequently discussed sources of support. Existing community provided the following types of support: emotional support (8.47% of all emotional support codes, 32/378), social companionship (7.04% of all social companionship codes, 14/199), instrumental support (15.22% of all instrumental support codes, 7/46), and informational support (1.56% of all informational support codes, 4/256). Cancer community provided the following types of support: social companionship (12.06% of all social companionship codes, 24/199), emotional support (5.82% of all emotional support codes, 22/378), informational support (0.78% of all informational support codes, 2/256), and instrumental support (2.17% of all instrumental support codes, 1/46). Existing community and cancer community provided similar types of support – emotional support and social support. Existing community, when discussed, consisted of spiritual and religious communities, sports communities, video-gaming communities, and work communities. Some AYAs described attending Bible study and church service and knowing that “they’re all praying for me, and that means the world to me”. One AYA described participating in sports with a national organization that supports individuals with health conditions. Cancer community, when discussed, consisted of cancer support groups and connections made with other pediatric cancer patients during inpatient stays. AYAs described the value in talking to peers with cancer who understood their experiences and to “know you’re not the only person going through it”.

### Global sufficiency of support

Coders (NL, KSB) demonstrated 100% agreement in global Sufficiency of Support codes prior to consensus conversations. We coded 11/32 (66%) participants as having sufficient social support. We coded 21/32 (34%) of participants as having mixed levels of social support, meaning that roughly one-third of our sample expressed feeling unsupported, dissatisfied with, or an absence of support in some domains. With peers, AYAs described difficulties maintaining social relationships, not being able to see their friends, and feeling left behind in reaching certain developmental milestones (e.g., going to college, getting a job). One AYA said “I used to have a lot of friends. I have like two now. …. I just feel behind and I feel sad sometimes that I’ve lost something.” (AYA-5014, 17 year-old, female) Another said “Each time I want to start college I relapse, right before school starts. And it's tough because I want to be with my friends, and they’re off going to college, and I guess, be with them at the same level, but unfortunately I have to attend [to] my health, which is the most important thing at this moment.” (AYA-5005, 19 year-old, male) AYAs also expressed being treated differently because they have cancer, with one stating “People seem to be, like acting differently around me … It felt like they were kind of pitying me. Of course, obviously I wouldn’t like that. But you can’t really just tell them to stop.” (AYA-5018, 17 year-old, male). Several AYAs voiced that cancer can be a lonely and isolating experience because “it’s hard to rant to someone about all this when they have never been through it” and “they just don’t understand”. They also described wanting to connect with other AYAs with cancer and having limited opportunities to do so, that “People with cancer exactly understand. … They can just laugh and you just know, because there are just so many things that only people with cancer have experienced.” (AYA-5014, 17 year-old, female).

With regard to family, AYAs frequently described parents and other caregivers as their primary and oftentimes sole source of support. AYAs described feeling guilty about the impact of cancer on their loved ones and worries about caregiver burden: “I’ve seen my parents go through it, where your child is having to go through something as horrendous as cancer and there’s nothing you can do about it. That, I’m sure, must be one of the worst feelings in the world.” (AYA-5028, 19 year-old, male) Similarly, another AYA described worrying about their mom, and “worry that it’s a lot of work for her, how much she’s doing, and how she’s feeling” even though their mom responds “it’s her job to help me, and that I don’t need to worry.” (AYA-5010, 14 year-old, male) Another aptly summarized, “Cancer, it’s not what it does to you but it’s what it does to the people around you.” (AYA-5019, 18 year-old, male).

## Discussion

The current study builds on emerging research about social support resources among AYAs with advanced cancer, and characterizes the support provided by family, the medical team, healthy peers, cancer peers, and the community. Previous literature has described the importance of social support for individual coping, mental health, and quality of life in AYAs with cancer [12, 13]. Only one previous study has examined social support in YAs with advanced cancer and found that greater perceived social support is associated with better psychosocial outcomes [18]. Consistent with other studies among AYAs with cancer, AYAs with advanced cancer who participated in our study described multiple sources of social support; family members were the primary source of all types of social support regardless of the AYA’s age; the medical team provided informational and emotional support; and, cancer peers provided informational and emotional support [11, 12]. Our study highlights the importance and impact of caregiver support as a major strength, but also suggests that there are opportunities for: expanding AYAs’ social support networks so that they are not solely reliant on caregivers alone; and, understanding caregiver burden and burnout and caregivers’ social support needs.

In our study, emotional support was provided the most frequently and by all sources of support. An overwhelming majority of participants described that family (namely, parents) were their primary source of support and provided all types of social support – emotional, informational, instrumental, and social companionship. Clinicians were commonly described as providing informational and emotional support, and healthy peers provided emotional support and social companionship. Although cancer service organizations (e.g., Livestrong, CancerCare, American Cancer Society) and previous studies emphasize the need for cancer support groups, there was less mention overall of cancer peers and existing community in our study; in our sample, this may be due to a relative lack of having received such support rather than not perceiving a need for social connection with cancer peers [31–33]. Where described, both sources of support provided emotional support and social companionship; those who had connected with other AYAs with cancer appreciated being able to talk to peers who could relate to their illness experience. Roughly one-third of the sample did not receive sufficient social support which is consistent with a previous systematic review that found that social support was among the most commonly expressed needs among AYA cancer survivors [34]. Our analysis showed that lack of support included AYAs referring to their parents as their sole source of support, and worrying about their parents’ mental health and well-being due to caregiver burden. Others described the loneliness and isolation of the cancer experience. AYAs expressed difficulties maintaining peer relationships and making new friends. Some participants discussed wanting to connect with cancer peers who can understand and relate to their illness experiences and having limited opportunities to do so.

The findings of the current study need to be interpreted with several limitations. First, all participants were recruited from a single pediatric academic medical center. Second, we had limited racial diversity in our sample which is representative of the Pacific Northwest setting in which our study was conducted but may limit the generalizability of our findings. Third, few of the AYAs described support provided by a romantic partner which is a source of support that has not yet been well-explored. Fourth, our findings may not reflect the experience of older young adults treated at adult cancer centers. Fifth, we were  only able to synthesize data and formulate conclusions based on what AYAs were willing to share which is an inherent limitation of studies relying on patient self-report. Sixth, social support is dynamic and this cross-sectional study characterizes AYAs’ perceptions of their social support at the time of the interview which was conducted pre-COVID-19 pandemic. Since the COVID-19 pandemic, young people have relied even more heavily on technology for online communication [35]. Despite this recent cultural shift, digital technology use for virtual support (i.e., social media, video games) was already a major focus of participants in the “Exploring the Concept of a ‘Good Death’ Study” [19]. Seventh, as this was a secondary analysis and not the primary objective of the original study, the interview guide did not explicitly query sources and types of support, or whether and how specific sources/types of support were lacking. Thus, our conclusions are constrained by the interview guide and the order in which questions were asked. Nonetheless, sources and types of support were organically discussed by all AYAs who participated in this study. Finally, given the small sample size of this study we were not powered for mixed-methods analyses to examine whether demographic characteristics (e.g., age, gender, socioeconomic status) were associated with differences in social support or psychosocial outcomes. However, our study included an appropriate sample size and rigorous research methods for qualitative research.

## Conclusions

AYAs with advanced cancer perceived caregivers as a universal source of support for all of their social support needs. They also described the importance of specific types of social support received from clinicians and peers. One-third of AYAs described receiving mixed levels of social support, reporting feeling unsupported, dissatisfied, or an absence of support in some domains. Future studies should granularly examine insufficient social support with regards to support sources and types, contributing factors that impact lack of perceived support, and in relationship to psychosocial outcomes. Future studies should also examine changes in social support resources and social support needs over time in AYAs with advanced cancer, and those navigating end-of-life decision making. Incorporating social support in palliative and supportive care interventions is a burgeoning area of research in individuals with life-limiting conditions [36, 37]. Further research should examine: palliative and supportive care interventions for increasing social support and communication in AYAs with advanced cancer; expanding social support resources beyond that which is provided by parents; opportunities to connect with other AYAs with cancer; and, strategies to stay connected with friends even when relocating for treatment. In addition, as families are the main source of support and AYAs reported on worries surrounding caregiver stress and burden, caregiver and family-based palliative and supportive care interventions are an important research priority. This can build on existing evidence-based interventions such as the Promoting Resilience in Stress Management (PRISM) intervention for AYAs [38, 39] and the Promoting Resilience in Stress Management for Parents (PRISM-P) [40, 41]. Continued exploration of opportunities to provide psychosocial support to families as a whole will lead to better individual- and family-based coping.

### Supplementary Information

Supplementary Material 1.

## Data Availability

The data used in the research cannot be publicly posted and cannot be shared privately with any person because participants did not consent to data sharing. This qualitative study was conducted with minors and in a geographically specific population. Any shared data, even if deidentified, may result in breach of confidentiality.

## Abbreviation

AYA: Adolescents and young adults
