# Supplementary material for: Social support resources in adolescents and young adults with advanced cancer: a qualitative analysis
Source: BMC Palliat Care. 2024 Jul 31;23:193. doi: 10.1186/s12904-024-01527-y (PMC11290203; doi:10.1186/s12904-024-01527-y)
Supplement: Supplementary file 1 — Supplementary Material 1. [file 12904_2024_1527_MOESM1_ESM.docx]

**Interview Guide – AYAs with Advanced Cancer**

[*Prior to recording*]

Thank you for agreeing to participate in this project. Before we begin, I’d like to review a few important points:

1. Please say exactly what you think. Don’t worry about what I think. This is about learning from your perspectives and experiences.
2. With that in mind, talk about your experience and feelings, not about what you have heard others say about this issue. Please express your opinions freely.
3. This conversation will be taped, so please do speak up and speak clearly.
4. Any reference to individual people or patients by name will be deleted from transcripts so that no identifiers are left in our research records. That said, identifying information will still remain on the audio file, which will be kept confidential until destroyed. Audio-recordings will be destroyed once analysis of all interview transcripts is completed.

Do you have any questions before we begin?

[*Begin Recording*]

The purpose of this project is to understand your experience, needs and perspectives as a teen or young adult with advanced cancer. To start, would you mind sharing with me, as best you can, what this experience has been like for you?

[*Probe Questions*]

I’m hoping we can talk today about when you learned that the cancer [hadn’t responded the way your doctors had hoped/had come back/was an aggressive type]. Can you share how that happened? (Whom did you hear from, how did you know, etc.)

*1) Did you talk to your parents about what is happening?*

If so:

Can you tell me more about that?

What is it like for you to have these conversations?

Can you tell me about the conversation(s)?

What is the hardest part about having these conversations?

What makes talking about this easier/better?

What if anything does your parent say that helps you?

If not:

Can you tell me more about that?

How do you feel about not talking about it?

Is there anything you wish you were talking about? If so, what?

What stops you from talking to your parent about the cancer?

How do you want your parents to share information with you about the cancer?

What information do you want about the cancer that you still don’t have?

*2) And when you think about the past month or so, has the way you talk about this (or not) within your family changed at all?*

If so: can you tell me more about that (repeat probes above)

If not: can you tell me more about that (repeat probes above)

*3) Who has helped you the most during this time?*

*(Probes) Is there anything that you feel like [support person] says or does that is particularly helpful?*

*Who else supports you?*

*Are there any supports/conversations/information you feel like you do not have and would like to have?*

*4) If you have questions about your cancer where do you go?*

*5) What worried/worries you most?*

*6) What things do you notice about your parent, if anything that causes you worry or concern?*

*7) When things get hard, what helps you most?*

*8) Culture often plays a big role in our world. Are there cultural influences that helped you or not in your experience with cancer?*

Thank you for discussing these questions with me today. I have one last question: If you were going to give advice to another teen or young adult with advanced cancer what advice would you give?

Before we close, do you have any questions for me or anything else you would like to talk about?

Do you have a support person, group or community to reach out to if you feel anxious or sad?

- Are there any additional resources that I can provide for you at this time?
